# Notochordal cell matrix: An inhibitor of neurite and blood vessel growth?

**DOI:** 10.1002/jor.24114

**Published:** 2018-08-06

**Authors:** Stefan A.H. de Vries, Marina van Doeselaar, Björn P. Meij, Marianna A. Tryfonidou, Keita Ito

**Affiliations:** ^1^ Orthopaedic Biomechanics, Department of Biomedical Engineering Eindhoven University of Technology P.O. Box 513 Eindhoven the Netherlands; ^2^ Faculty of Veterinary Medicine, Department of Clinical Sciences of Companion Animals Utrecht University Utrecht the Netherlands; ^3^ Department of Orthopaedics University Medical Center Utrecht the Netherlands

**Keywords:** notochordal cells, intervertebral disc, angiogenesis, neurogenesis

## Abstract

Blood vessel and neurite ingrowth into the degenerating intervertebral disc (IVD) are related to pain. In reported studies, notochordal cell (NC)‐conditioned medium (NCCM) induced a regenerative response of nucleus pulposus (NP) cells, but also inhibition of neurite and vessel formation. NC matrix (NCM) derived from NC‐rich NP tissue, induced even stronger anabolic effects than NCCM. Thus, the aim was to investigate whether NCM has similar anti‐neurogenic and ‐angiogenic properties as NCCM. NCM and NCCM where produced from porcine NC‐rich NP tissue. Human umbilical vein endothelial cells (HUVECs) were cultured in base medium (BM, 300 mOsm), NCCM (produced at 300 and 400 mOsm), NCM, or with chondroitin sulfate (CS, positive control) in angiogenesis‐inducing medium, after which vessel length was measured. Although CS alone inhibited vessel growth, NCCM (both osmolarities) stimulated vessel formation by HUVECs. NCM did not affect vessel growth relative to BM. SH‐SY5Y cells were cultured in BM, NCCM, and NCM on poly‐D‐lysine coated and polystyrene surfaces, and analyzed for neurite length and percentage of neurite expressing cells. On coated surfaces, neither NCCM nor NCM affected neurite growth. On a polystyrene surface, NCCM and NCM induced a higher number of neurite‐expressing cells. NCCM's previously reported anti‐angiogenic and ‐neurogenic effects were not observed in this study. Although addition of CS inhibited HUVEC vessel formation, other factors may be present in NCCM and NCM that affect neurite and vessel growth. Therefore, future studies testing an NC‐based regenerative strategy should carefully assess the risk of such adverse effects in an in vivo setting. © 2018 The Authors. *Journal of Orthopaedic Research®* Published by Wiley Periodicals, Inc. J Orthop Res 36:3188–3195, 2018.

Intervertebral discs (IVDs) are the structures in between the vertebrae that transmit loads and provide flexibility to the spine. The IVD consists of a hydrated core, the nucleus pulposus (NP), which is circumferentially confined by the annulus fibrosus (AF). Negatively charged glycosaminoglycans (GAGs), bound within proteoglycans (PGs), indirectly attract water into the NP. Thereby the swelling pressure inside the NP rises, which is crucial for sustaining high compressive loads. During IVD degeneration, the swelling pressure decreases due to a loss of PGs, and compressive loads are increasingly exerted on the AF, possibly leading to annular tears and further degenerative changes.

Annular tears, which are more frequently found with increasing age,^1^ are associated with low back pain.^2^, ^3^ Whereas the healthy IVD is mostly aneural and avascular, annular tears provide a low pressure micro‐environment, and are therefore suggested to serve as entry‐portal for nerve and blood vessel ingrowth into the degenerated NP.^4^ Indeed, in elderly discs, many tears showed ingrowth of nerves, often accompanied by blood vessels.^5^ Furthermore, the site of neurite and blood vessel ingrowth has been found to correspond to the levels where patients experienced pain,^6^ suggesting a direct relation between IVD nerve ingrowth and low back pain.

Previously, nerve growth factor (NGF) was suggested as the driving factor for nerve ingrowth in the IVD.^7^ The study by Freemont et al. also showed that microvessels are a source of NGF, suggesting that an interplay between nerves and blood vessels is responsible for the innervation of the painful disc. In addition to NGF, vascular endothelial growth factor (VEGF) and brain‐derived neurotrophic factor (BDNF) are also believed to be involved with neurite and blood vessel growth in the IVD. Cells from the human NP and AF are able to produce these factors, and stimulation with inflammatory cytokines upregulated their production.^8^, ^9^, ^10^ Altogether this suggests that the degenerated IVD, which is characterized by the presence of inflammatory cytokines in combination with a low osmotic environment due to proteoglycan loss, may facilitate nerve and blood vessel ingrowth.

Notochordal cells (NCs), and more specifically NC‐conditioned medium (NCCM), has received considerable attention due to its IVD regenerative potential. NCCM has been produced by incubating either isolated NCs or NC‐rich NP tissue in culture medium for several days. The factors secreted by NCs induced an anabolic response in vitro of nucleus pulposus cells (NPCs) and bone marrow‐derived mesenchymal stem cells (BMSCs) in various animal models.^11^, ^12^, ^13^, ^14^, ^15^, ^16^, ^17^, ^18^, ^19^ Furthermore, NCs and IVD matrix proteins have been investigated with regard to anti‐angiogenic and ‐neurogenic properties. Human IVD aggrecan has shown to inhibit endothelial cell migration.^20^ Presence of NCs and NPCs inhibited neurite outgrowth from dorsal root ganglion explants,^21^, ^22^ and angiogenic potential of endothelial cells.^23^ Also NCCM has been shown to inhibit neurite growth by human neuroblastoma (SH‐SY5Y) cells^24^ and blood vessel formation by human umbilical vein endothelial cells (HUVECs).^25^ GAGs in NCCM such as chondroitin sulfate (CS) were found to be responsible for the inhibitory effect, since chondroitinase‐treated NCCM did not inhibit neurite and vessel growth. Therefore, besides induction of an anabolic response, a NC‐based regenerative therapy was suggested to also counteract low back pain by inhibiting neurite and blood vessel growth.

Recently, we proposed an alternative to NCCM, that is, natural NC‐derived matrix (NCM), which can be produced by lyophilizing and pulverizing porcine NP tissue that was mostly populated by NCs. By using NCM, the NCCM incubation period, in which bioactive factors may be subject to degradation and inflammatory factors may be produced as a result of free swelling conditions^26^ is omitted. NCM induced stronger proteoglycan production and proliferation of bovine NPCs compared to NCCM,^27^ and induced a regenerative and anti‐inflammatory effect in a canine in vivo model^28^ underscoring its potential in biological IVD repair. Furthermore, NCM is directly harvested from the healthy IVD, hence it is also rich in GAGs. As such, we hypothesize that NCM also inhibits neurite growth and vessel formation, similar to NCCM.^24^, ^25^ Therefore, as a first step to determine the anti‐neurogenic and ‐angiogenic potential of NCM, the aim of this study was to test whether NCM inhibits HUVEC tubule formation and SH‐SY5Y neurite growth, similar to NCCM. Moreover, NCCM has previously been produced in different osmotic conditions, often by incubation in basal culture medium, but also in medium with increased osmolarity to provide a more physiological environment. Thus, we also tested whether the osmotic conditions during NCCM production would affect its anti‐angiogenic properties.

## MATERIALS AND METHODS

### Generation of NCCM and NCM

NC‐rich NP tissue was harvested within 6 h after slaughtering from the cervical, thoracic, and lumbar IVDs of five young (approximately 3‐month‐old) pigs, obtained from the local abattoir. The IVDs were opened aseptically, and the NP was taken out, careful not to include AF tissue. To produce NCCM, half of the NP tissue from each spine was incubated in high glucose Dulbecco's modified eagle's medium (hgDMEM, Invitrogen, Carlsbad, CA) supplemented with 1% penicillin/streptomycin (P/S, Lonza, Basel, Switzerland), 1× insulin transferrin selenium (ITS‐1^+^, BD Biosciences, Breda, the Netherlands) and 50 μg/ml ascorbic acid‐2‐phosphate (Sigma, Zwijndrecht, the Netherlands) at 30 ml medium per gram of tissue as described previously.^18^ The NP tissue was incubated for 4 days at 37°C, 5% CO_2_ and 5% O_2_ after which the NP tissue was removed from the medium by filtration with a 70 μm pore size cell strainer. Subsequently, the medium was filtered through a 3 kDa cut‐off filter tube (Amicon Ultra‐15 centrifugal filter, Merck, Darmstadt, Germany), and the strained material was resuspended in the same amount of fresh culture medium. For HUVEC culture, this material was resuspended in Medium 200 (Life Technologies, Bleiswijk, the Netherlands). For SH‐SY5Y cell culture, the strained material was resuspended in a 1:1 mixture of Eagle's minimum essential medium (EMEM) and F‐12 K medium (both from Life Technologies). To produce NCM, the other half of the NP tissue of each spine was lyophilized overnight and pulverized to a fine powder using a microdismembrator (Sartorius, Goetingen, Germany). NCCM and NCM (*n* = 5 donors) were aliquoted and stored at −80°C until further use.

### Biochemical Analysis of NCCM and NCM

NCCM and NCM from five different porcine donors were analyzed for GAG and protein content, in order to apply them in a similar concentration and to be able to make appropriate comparisons. To determine GAG concentration, samples were digested overnight at 60°C in papain solution (100 mM phosphate buffer, 5 mM l‐cysteine, 5 mM ethylene diamine tetra‐acetic acid, and 140 mg/ml papain; all from Sigma). A dimethyl methylene blue (DMMB) assay was performed,^29^ using shark cartilage chondroitin sulfate (Sigma) as a reference curve. NCCM and NCM protein content were determined using a bicinchinonic acid (BCA) assay with a reference curve from bovine serum albumin (BSA).

NCCM contained 902 ± 112 μg GAG/ml, whereas NCM contained 472 ± 22 μg GAG/mg NP tissue (average and standard deviation from five different donors). Therefore, for NCM cultures approximately 2 mg NCM was dissolved in 1 ml BM, to apply a similar GAG concentration between NCCM and NCM. The protein concentration in NCCM was 614 ± 69 μg/ml and that for NCM was 290 ± 13 μg/mg (average and standard deviation from five different donors). Hence, normalization for GAG content, resulted in an approximately similar protein concentration between NCCM and NCM employed in the culture experiments.

### HUVEC Vessel Formation With NCCM and NCM

HUVECs (Life Technologies, pool of 10 donors) were expanded in basal medium (BM, 300 mOsm) consisting of Medium 200 supplemented with low serum growth factors (LSGS, Life Technologies) and 1% P/S until 80% confluency. A 24‐well plates were coated with 120 μl/well Geltrex LDEV‐free (Thermo Fisher Scientific, Breda, the Netherlands), after which HUVECs at passage two were seeded at 80,000 cells per well. Cells were incubated for 24 h at 37°C and 5% CO_2_ in three different medium conditions: BM, NCCM with the same supplements as BM or NCM added to BM (*n* = 5 biological replicates for each condition). The NCM concentration in medium was adjusted to obtain similar GAG content to NCCM. After 24 h, cells were incubated for 15 min with 10 μM Calcein AM and 10 μM propidium iodide. Per well, three pictures were taken at 4× magnification using a fluorescence microscope (Zeiss Axiovert, Zeiss, Jena, Germany). Per sample, the images were taken at approximately the same locations, covering the largest part of the well without overlap. To assess whether effects of NCCM or NCM may be dependent on HUVEC donor variation, the same experiment was repeated with HUVECs (used at passage 2) from two separate additional donors (Cell Applications, San Diego, CA, *n* = 2 biological replicates each).

### HUVEC Vessel Formation in Basal and Physiological Osmolarity NCCM

Since osmolarity changes are known to affect NC phenotype,^30^ the effect of NCCM produced at different osmolarities on HUVEC vessel formation was tested. The experiment was performed as described previously, with different medium groups: BM (300 mOsm), NCCM produced at basal and physiological osmolarity (300 and 400 mOsm, respectively) NCCM (*n* = 5 biological repeats for each medium condition). Physiological osmolarity NCCM was produced similar to basal NCCM, but with addition of a 1% salt solution (5 M NaCl/0.4 M KCl) to the medium during the 4 day incubation period, increasing medium osmolarity by 108 mOsm as previously described.^25^ In these experiments, also a chondroitin sulfate (CS, Sigma, Zwijndrecht, the Netherlands) group was included, where an amount of CS equivalent to the GAG concentration in NCCM (approximately 900 μg/ml) was added, as a control for the inhibitory effect of CS itself.

### SH‐SY5Y Cell Neurite Growth

SH‐SY5Y cells (Sigma) were cultured up to 70% confluency in EMEM:F‐12 K (1:1) supplemented with 10% fetal calf serum (FCS, Gibco: Invitrogen, Carlsbad, CA) and 1% P/S. Subsequently, cells were seeded at a density of 50,000 cells per well in a poly‐D‐lysine coated six‐well plate that had been employed before for similar experiments exploring the anti‐neurogenic effects of NCCM.^24^ SH‐SY5Y cells were incubated for 24 h at 37°C and 5% CO_2_ in expansion medium to allow them to attach. After 24 h, medium was aspirated and the wells were washed twice with phosphate buffered saline (PBS, Sigma) to remove serum. Thereafter, wells were divided into 4 different medium conditions: Basal medium (BM: EMEM:F‐12 K (1:1) supplemented with 1% P/S), positive control medium (BM + PDGF: BM supplemented with 40 ng/ml platelet derived growth factors (PDGF, R&D Systems, Abingdon, UK) to induce neurite growth, NCCM + PDGF and NCM + PDGF (*n* = 5 biological repeats for each condition). NCCM and NCM contained the same supplements as BM + PDGF. The NCM concentration in medium was adjusted to obtain similar GAG content between NCCM and NCM. Cells were incubated for 48 h at 37°C and 5% CO_2_. After 48 h, 10 phase contrast images per well were taken at 10× magnification using a bright‐field microscope (Zeiss Observer, Zeiss, Jena, Germany). The images were taken at random location, ensuring no overlap. To determine the effect of the culture surface on which SH‐SY5Y cells were incubated, this experiment was repeated (*n* = 4 biological repeats for each condition) with polystyrene six‐well plates.

### Image Analysis

HUVEC vessel length as well as SH‐SY5Y cell neurite length were measured using ImageJ Simple Neurite Tracer. For HUVECs, only the vessels themselves were measured, not taking into account the branch points. To ensure that actual vessels were measured, only processes larger than 50 μm were taken into account. The total vessel length from three pictures per well was expressed per surface area. For SH‐SY5Y cells, only cells and neurites that were in the field of view in their entirety were counted and measured. For branching neurites, only the longest branch was measured, and only neurites longer than 20 μm were taken into account. From this, the percentage of neurite‐expressing cells and average neurite length were calculated.

### Statistics

Statistical analysis was performed with Statistical Package for Social Sciences (SPSS, version 22; IBM, Armonk, NY). Normality was tested using the Shapiro–Wilk test. Normally distributed data were analyzed using a one‐way analysis of variance (ANOVA), followed by independent *t*‐test post hoc testing with Bonferroni corrections. For non‐normally distributed data, a Kruskal–Wallis test was used, followed by Mann–Whitney U tests with post‐hoc Bonferroni corrections. Statistical significance was accepted for *P* values <0.05.

## RESULTS

### HUVEC Vessel Formation With NCCM and NCM

**Figure 1:**
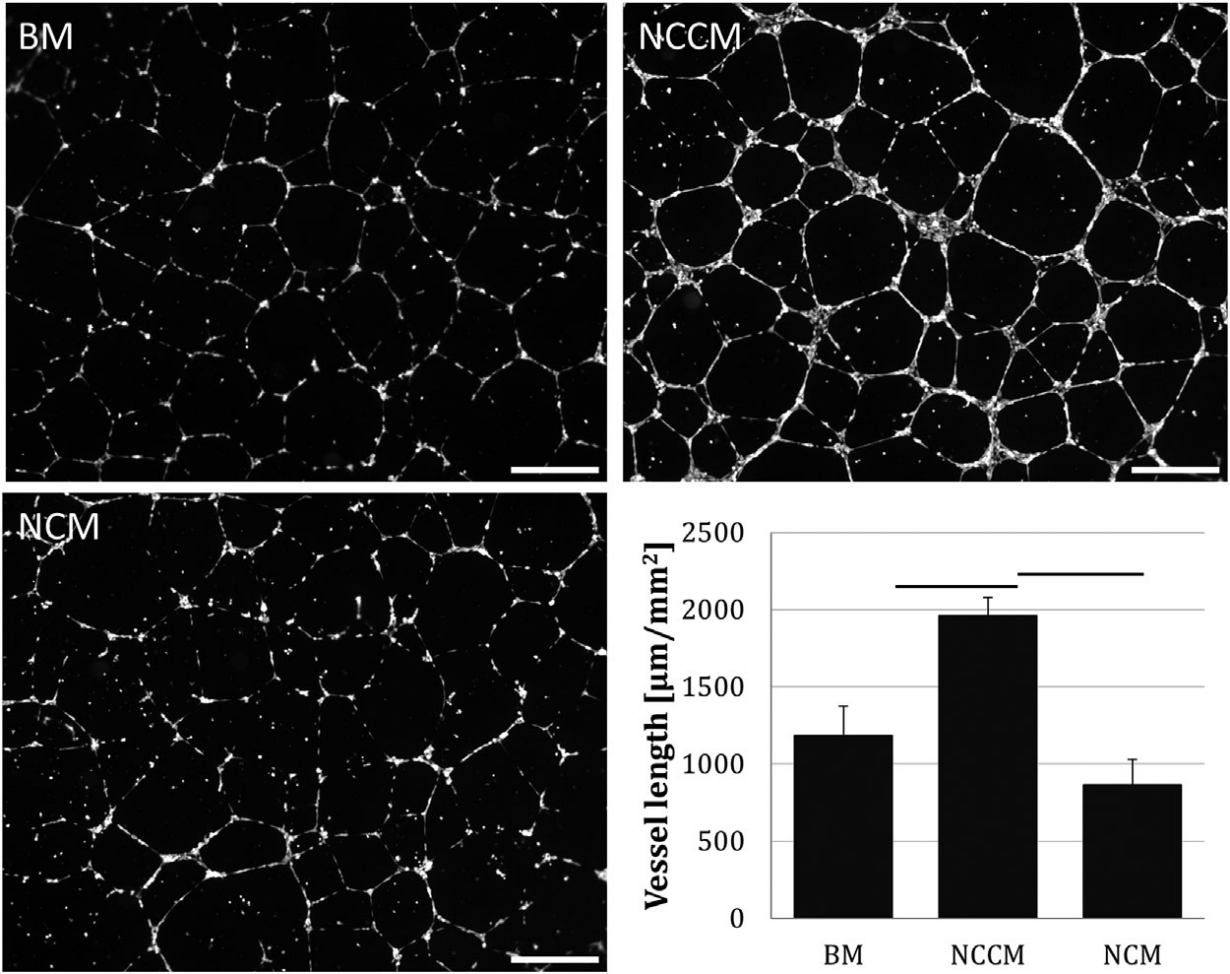
Representative images of human umbilical vein endothelial cell (HUVEC) vessel formation and vessel length measurements in base medium (BM), porcine notochordal cell‐conditioned medium (NCCM), and porcine notochordal cell‐rich matrix (NCM). Scale bars represent 250 μm. Values represent means + standard deviations, *n* = 5 biological repeats. Bars indicate *P* < 0.05 between indicated groups.

After 24 h of incubation, only limited cell death was observed, with little difference between experimental groups. Vessel formation was observed in all groups (Fig. 1). However, in NCCM, the vessel network appeared more matured and organized compared to BM, where vessels were more often thinner and interrupted. In NCM, vessel formation was not uniform, as vessels were produced in some areas, whereas other areas hardly showed vascular organization. Vessel length was significantly higher with NCCM compared to BM and NCM. No significant difference in vessel length was observed between BM and NCM.

To assess whether this finding could be due to HUVEC donor variation in response to NCCM and NCM, HUVECs from two additional donors were subjected to the same experiment (*n* = 2 biological repeats per NCCM/NCM donor). Although there were donor differences in total vessel length, the overall pattern between culture groups was similar to the initial experiment; vessel length increased with NCCM (donor 1: 1212 and 1235 μm/mm^2^, donor 2: 1431 and 1527 μm/mm^2^) compared to BM (donor 1: 2187 and 2218 μm/mm^2^, donor 2: 2440 and 2401 μm/mm^2^), whereas it was unaffected by NCM (donor 1: 998 and 957 μm/mm^2^, donor 2: 1468 and 1593 μm/mm^2^), similar as observed with the pooled HUVECs of the initial experiment.

### HUVEC Vessel Formation in Basal and Physiological Osmolarity NCCM

**Figure 2:**
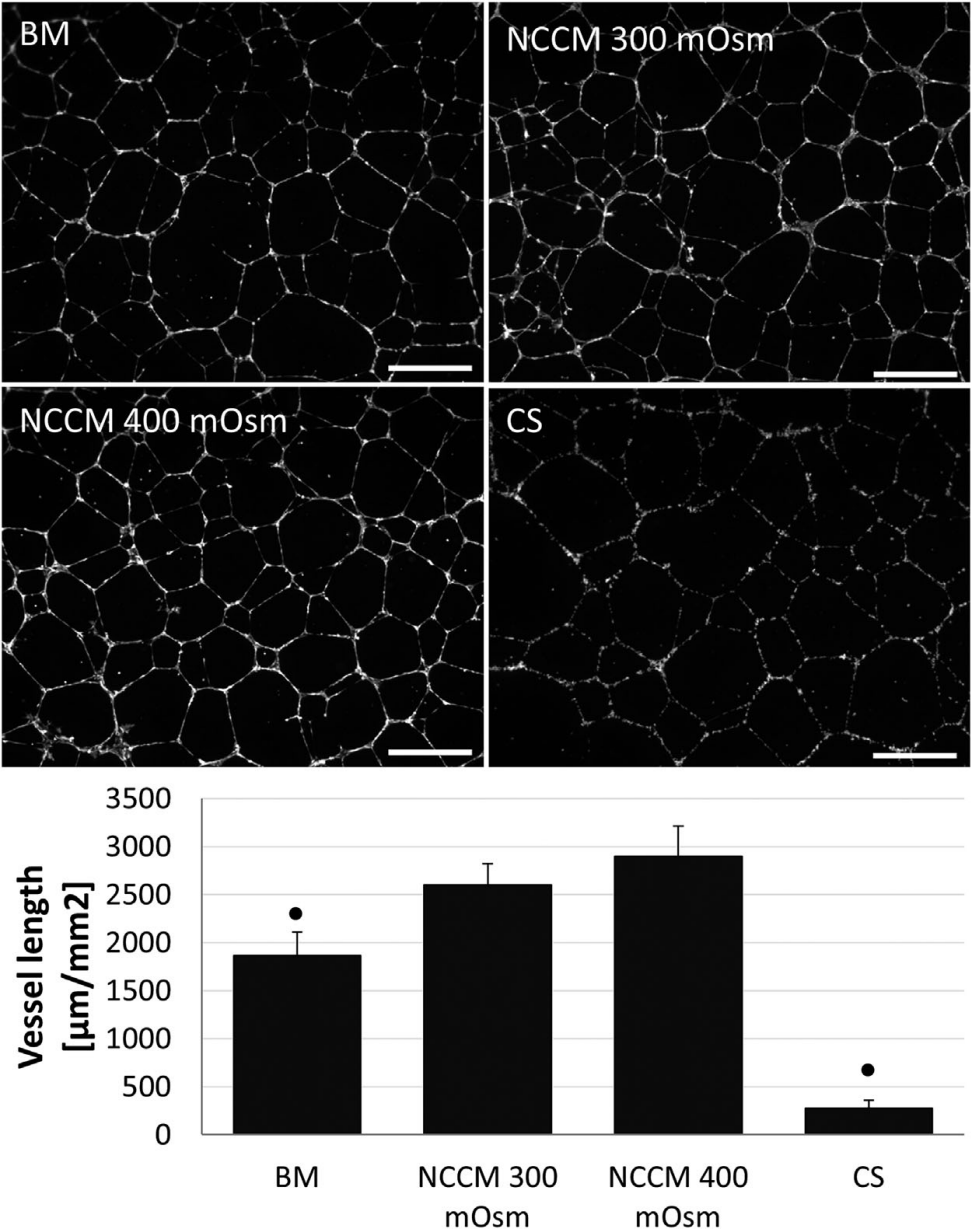
Representative images of human umbilical vein endothelial cell (HUVEC) vessel formation and vessel length measurements in base medium (BM, 300 mOsm), porcine notochordal cell‐conditioned medium (NCCM) produced at approximately 300 and 400 mOsm, and with addition of an equivalent amount of chondroitin sulphate (CS). Scale bars represent 250 μm. Values represent means + standard deviations, *n* = 5 biological replicates. ● indicates *P* < 0.05 compared to all other groups.

Given that previous experiments exploring the anti‐angiogenic properties of NCCM where conducted with NCCM generated at 400 mOsm,^25^ we explored whether extracellular osmolarity affected NCCM's angiogenic properties. For this purpose, HUVEC vessel formation was measured after culture in NCCM produced at approximately 300 and 400 mOsm, with an additional positive control, that is, a culture condition including CS. NCCM at both osmolarities significantly increased the total vessel length compared to BM (at 300 mOsm) with no differences between the two types of NCCM (Fig. 2). Addition of CS alone resulted in a significantly decreased vessel length compared to all other culture groups. In the presence of CS, the vessel network could be distinguished but HUVECs appeared as single cells with only sporadic and small vessel outgrowths (Fig. 2).

### SH‐SY5Y Cell Neurite Growth

**Figure 3:**
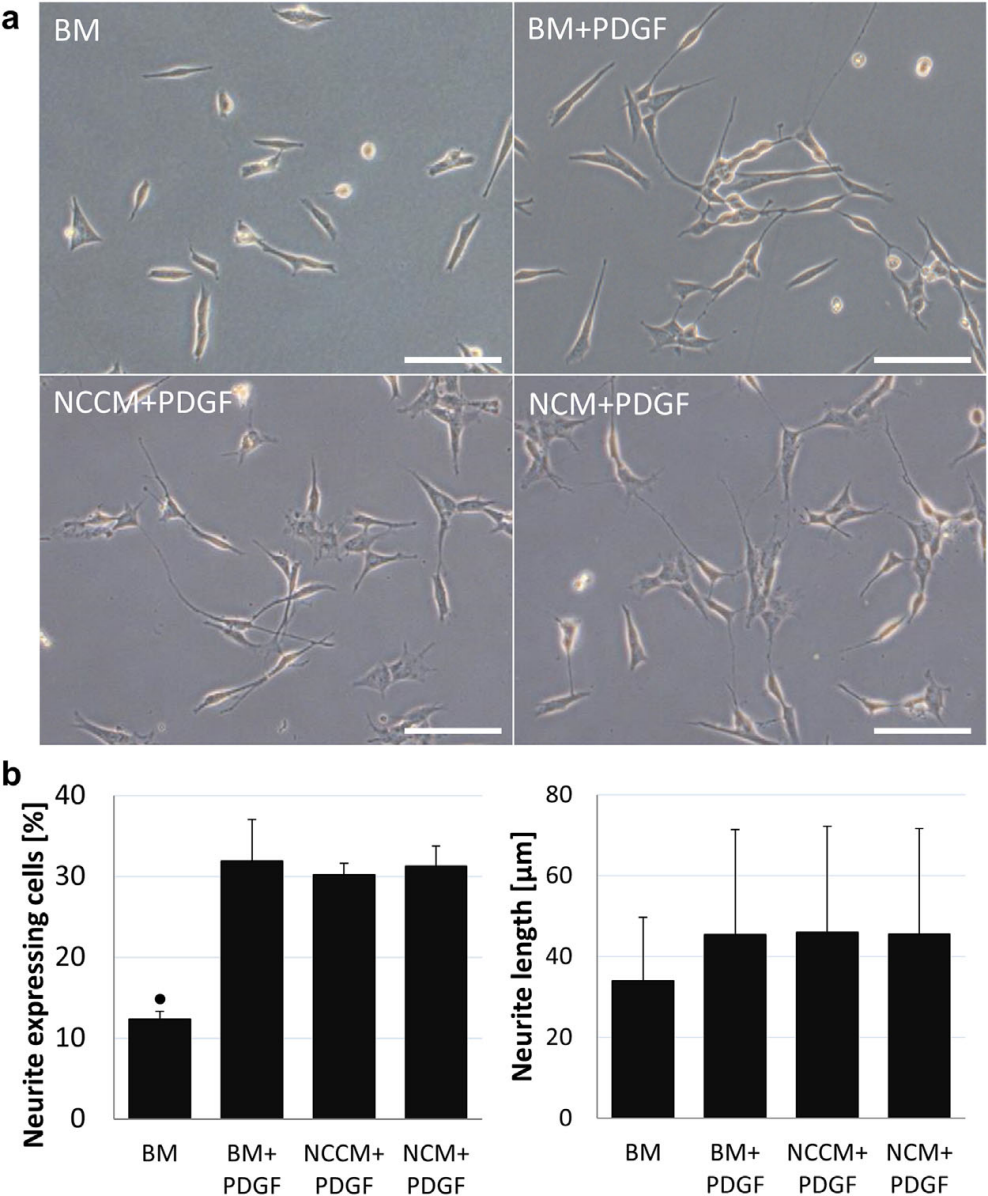
(a) Human neuroblastoma (SH‐SY5Y) cell neurite growth on a poly‐D‐lysine coated surface in base medium (BM), BM with platelet derived growth factors (BM + PDGF), porcine notochordal cell conditioned medium (NCCM) supplemented with PDGF (NCCM + PDGF), and porcine NC‐rich nucleus pulposus matrix (NCM) supplemented with PDGF (NCM + PDGF). Scale bars represent 100 μm. (b) Percentage of neurite expressing cells and average neurite length. Values represent means + standard deviations, *n* = 5 biological replicates. ● indicates *P* < 0.05 compared to all other groups.

**Figure 4:**
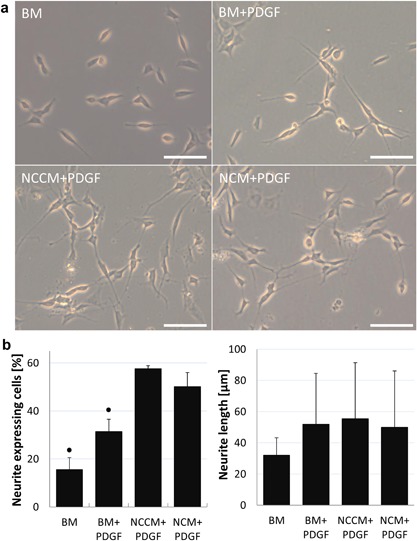
(a) Human neuroblastoma (SH‐SY5Y) cell neurite growth on a polystyrene surface in base medium (BM), BM with platelet derived growth factors (BM + PDGF), porcine notochordal cell conditioned medium (NCCM) supplemented with PDGF (NCCM + PDGF), and porcine NC‐rich nucleus pulposus matrix (NCM) supplemented with PDGF (NCM + PDGF). Scale bars represent 100 μm (b) Percentage of neurite expressing cells and average neurite length. Values represent means + standard deviations, *n* = 4 biological repeats. ● indicates *P* < 0.05 compared to all other groups.

On a poly‐D‐lysine coated surface, the percentage of neurite expressing cells significantly increased in all PDGF‐treated groups compared to BM (Fig. 3a and b). The percentage of neurite‐expressing cells and average neurite length in NCCM + PDGF and NCM + PDGF was however not different from BM + PDGF, and no differences in neurite length where observed. To assess whether the culture surface influenced the effect of NCCM and NCM on SH‐SY5Y cell neurite growth, cells were also grown on polystyrene surfaces under the same conditions as before. Again, with BM + PDGF a higher number of neurite‐expressing cells compared to BM was observed (Fig. 4a and b) which further increased with NCCM + PDGF as well as with NCM + PDGF. Similar to poly‐D‐lysine coated surface, also on a polystyrene surface, no differences in neurite length where observed between medium conditions.

## DISCUSSION

NCM may have practical advantages over NCCM in a NC‐based treatment strategy for IVD degeneration. The aim of the current study was therefore to investigate whether NCM had a similar effect to NCCM in inhibiting vessel formation by HUVECs and neurite growth by SH‐SY5Y cells. Interestingly, contrary to others,^24^, ^25^ neither NCCM nor NCM significantly inhibited vessel formation and neurite growth in the present study. In fact, NCCM stimulated HUVEC vessel formation, and in the presence of PDGF, both NCCM and NCM stimulated SH‐SY5Y cell neurite growth further relative to BM on a polystyrene surface.

An important difference in production of NCCM and NCM is the 4 day incubation period for NCCM, which is omitted with NCM production. Possibly, angiogenic factors are produced by the NCs during NCCM incubation which may be responsible for the differences in vessel formation with NCCM and NCM. Furthermore, whereas in previous study NCCM was produced in medium with increased osmolarity,^25^ the initial experiment in the current study tested vessel formation with NCCM generated at basal (unadjusted) medium osmolarity. Extracellular osmolarity is known to regulate cell volume^31^, ^32^ and gene expression,^30^, ^33^ as expression of proposed NC‐markers brachyury and cytokeratin 18 was decreased in 300 mOsm culture medium compared to 400 mOsm. However, the current study revealed that also NCCM produced at approximately 400 mOsm did not inhibit HUVEC vessel formation. In fact, total vessel length increased with 400 mOsm NCCM compared to BM, similar as NCCM produced at basal osmolarity. This indicates that the difference in osmolarity at which NCCM is produced does not explain the different results between the current and previous study by Cornejo et al.

The finding that HUVEC vessel length increased with NCCM produced at both osmolarities, but not with addition of NCM, suggests that angiogenic factors may be produced during the 4 day incubation period to produce NCCM, irrespective of extracellular osmolarity. During generation of NCCM, NCs produce bioactive substances that are released into the medium, and at the same time the surrounding NP tissues is also subjected to degradation, releasing thereby matrix components. In line with this thought, semaphorins (SEMA3C, SEMA7A) have been detected in porcine NCCM.^34^, ^35^ SEMA3C plays a role in vascularization and innervation of IVDs and has been linked to low back pain,^36^ and may have stimulated vessel formation in the current study. However, semaphorins are a complex family of proteins that also display anti‐angiogenic effects,^37^ and have stimulating as well as inhibiting effects on nerve growth.^38^ Hence the effects of semaphorins in the current study remains elusive. The proteomic analysis of porcine NCCM did not detect (potentially) angiogenic factors other than semaphorins, probably due to their low abundance in respect to matrix protein. As such, it may still be possible that other factors such as vascular endothelial growth factor or inflammatory cytokines were present in the NCCM, and accounted for the observed angiogenic effects in the present study. The potential production of angiogenic factors may explain the difference in vessel formation between NCCM and NCM, since NCM is directly harvested from the IVD and lyophilized. Nonetheless, no significant decrease in vessel length was observed with NCM compared to BM indicating that NCM did not modulate angiogenesis. Presence of such angiogenic factors within the healthy NC‐rich NP tissue, together with the angiogenesis‐inhibiting GAGs in the NCM may have balanced promotion and inhibition of vessel formation, resulting in a similar vessel length between NCM and BM. The latter is further supported by the fact that CS significantly inhibited vessel length compared to BM.

The question still remains what causes the different results between the current study and that by Cornejo et al. HUVEC donor variation is not likely, as a stimulatory effect on vessel formation was observed for all three independent HUVEC donors tested in the current study. Furthermore, addition of CS to the HUVEC cultures had comparable inhibitory effects on vessel formation, indicating that the HUVECs do respond to proper anti‐angiogenic stimuli. The difference between studies is more likely NCCM‐related. NCCM was generated from NP tissue from 12‐week‐old pigs in our study, whereas NP tissue was harvested from 6‐ to 8‐week‐old pigs in the study by Cornejo et al. The small age difference may have contributed to the contradicting results. Additionally, it cannot be excluded that NCCM produced from NP tissue of different pig breeds has differential effects on HUVEC vessel formation. Furthermore, in previous studies that investigated NCCM's anti‐angiogenic and ‐neurogenic potential, NCCM was produced at 1% oxygen, whereas it was produced at 5% oxygen in the current study. Previous studies comparing NC behavior in normoxia (21% oxygen) and hypoxia (1–3.5% oxygen) found oxygen‐related differences in NC morphology, gene expression and lactate production,^39^, ^40^, ^41^ suggesting that NCs are sensitive to oxygen concentrations. As such, we cannot exclude the difference between NCCM production at 5% oxygen in the current study and 1% oxygen the previous studies could account for the differential effects. Altogether this indicates that the source of NP tissue and manufacturing process of NC‐derived technologies is of great importance when exploiting the full therapeutic potential of NC‐based strategies.

While NCCM has been reported to inhibit neurite growth by SH‐SY5Y cells,^24^ in the current study, neither NCCM nor NCM affected the percentage of neurite expressing cells or the average neurite length when SH‐SY5Y cells where grown on a poly‐D‐lysine coated surface. Even more so, both NCCM and NCM increased the percentage of neurite expressing cells compared to the positive control when cultured on a polystyrene culture surface. The latter implies the presence of neurogenic factors in NCCM and NCM. Furthermore, this finding suggests that the ability of SH‐SY5Y cells to grow neurites is affected by the culture surface. Neurons are routinely cultured on poly‐D‐lysine coated surfaces to promote cell attachment. However, from a range of coatings tested, poly‐D‐lysine inhibited proliferation of mesenchymal stem cells (MSCs), and showed the poorest neurite outgrowth of neurogenic differentiated MSCs.^42^ This could indicate that in the present study, neurite growth of SH‐SY5Y cells was hindered due to strong adhesion to the poly‐D‐lysine coated surface, whereas a weaker adhesion of the cells to a polystyrene surface facilitated the formation of neurites in the presence of neurogenic factors in NCCM and NCM. The higher percentage of neurite expressing cells with NCCM + PDGF as well as NCM + PDGF compared to BM and BM + PDGF suggests that neurogenic factors may already be present in the healthy NC‐rich IVD, and are not only produced by NCs during NCCM production.

In conclusion, application of a NC‐based regenerative treatment strategy should, in the first place, provide an anabolic response to the resident NPCs to restore the IVD to a healthy state. Ideally, concomitant inhibition of neurite and vessel ingrowth may contribute to pain management. Such an effect was however not established in the current study. In fact, although CS indeed inhibited vessel formation, other factors are present in NCCM and/or NCM that may affect blood vessel formation and neurite growth. Such adverse effects pose a potential risk of an NC‐based regenerative therapy and should be carefully evaluated in more physiologically relevant models, for example, using a chorioallantoic assay in the developing chick embryo for angiogenesis and dorsal root ganglia rather than the SH‐SY5Y cell line for evaluation of neurite growth.

## AUTHORS' CONTRIBUTIONS

SdV, MvD, and KI designed the HUVEC and SH‐SY5Y cell culture experiments. SdV prepared NCCM and NCM. SdV and MvD set up experiments and took neurite and vessel pictures. SdV analyzed vessel length, neurite length, and number of neurite‐expressing cells. SdV, MvD, MT, and KI interpreted these data. SdV prepared the manuscript, and all authors contributed and provided critical feedback. KI oversaw the study, and gave the final approval for the submitted version of the manuscript.
